# Cis-regulatory elements and transcription factors related to auxin signaling in the streptophyte algae *Klebsormidium nitens*

**DOI:** 10.1038/s41598-023-36500-x

**Published:** 2023-06-15

**Authors:** Noriaki Tounosu, Kanami Sesoko, Koichi Hori, Mie Shimojima, Hiroyuki Ohta

**Affiliations:** School of Life Science and Technology, Tokyo Institute of Technology, 4259 B-65, Nagatsuta-cho, Midori-ku, Yokohama, Kanagawa 226-8501 Japan

**Keywords:** Plant evolution, Auxin

## Abstract

The phytohormone auxin affects numerous processes in land plants. The central auxin signaling machinery, called the nuclear auxin pathway, is mediated by its pivotal receptor named TRANSPORT INHIBITOR RESPONSE 1/AUXIN SIGNALING F-BOX (TIR1/AFB). The nuclear auxin pathway is widely conserved in land plants, but auxin also accumulates in various algae. Although auxin affects the growth of several algae, the components that mediate auxin signaling have not been identified. We previously reported that exogenous auxin suppresses cell proliferation in the *Klebsormidium nitens* that is a member of streptophyte algae, a paraphyletic group sharing the common ancestor with land plants. Although *K. nitens* lacks TIR1/AFB, auxin affects the expression of numerous genes. Thus, elucidation of the mechanism of auxin-inducible gene expression in *K. nitens* would provide important insights into the evolution of auxin signaling. Here, we show that some motifs are enriched in the promoter sequences of auxin-inducible genes in *K. nitens*. We also found that the transcription factor KnRAV activates several auxin-inducible genes and directly binds the promoter of *KnLBD1*, a representative auxin-inducible gene. We propose that KnRAV has the potential to regulate auxin-responsive gene expression in *K. nitens*.

## Introduction

Phytohormones are essential signaling molecules for land plants to respond to developmental cues and adapt to environmental conditions during different phases of their life cycles^1–3^. The phytohormone auxin affects numerous aspects of plant development including embryogenesis^4,5^ and organogenesis^6,7^ as well as environmental adaptation such as tropic responses^8–10^. Decades of research with seed plants has revealed much about auxin signaling^11,12^. The central machinery that facilitates the cellular response to auxin starts with auxin perception by the receptor TIR1/AFB (transport inhibitor response 1/auxin signaling F-box). Non-covalent binding of auxin to TIR1/AFB mediates the interaction between this receptor and auxin/indole-3-acetic acid (Aux/IAA), a negative regulator of the auxin response, leading to ubiquitination of Aux/IAA via the Skp1-Cullin-F-box complex and subsequent degradation of Aux/IAA in the proteasome. When the concentration of all auxins is low, Aux/IAA directly interacts with the transcription factor ARF (auxin response factor) and inhibits its function; at high auxin concentration, the proteasomal degradation of Aux/IAA, mediated by TIR1/AFB, releases ARF from Aux/IAA, which allows ARF to activate transcription of downstream genes. This pivotal auxin signaling machinery, i.e., comprising TIR1/AFB, Aux/IAA, and ARF, is called the nuclear auxin pathway, which controls gene expression via sensing auxin concentration throughout the plant life cycle.

Comparative genomic analyses have indicated that the components of the nuclear auxin pathway are widely conserved in land plants^13^, including bryophytes, a sister group of vascular plants. The moss *Physcomitrium patens* was the first bryophyte lineage to have its genome sequenced^14,15^. Based on that genome, various studies revealed that multiple copies of the components of the nuclear auxin pathway are conserved and that those components contribute to the auxin response in *P. patens*^16^. Furthermore, the sequenced genome of the liverwort *Marchantia polymorpha* suggested that a much simpler auxin signaling system, i.e., with a single TIR1/AFB homolog, functions in this lineage^17–20^. Similarly, the hornwort *Anthoceros agrestis* has low numbers of the components of the nuclear auxin pathway^21^. These data support the idea that the nuclear auxin pathway is a well-established system that had already been acquired in the common ancestor of land plants^13^.

The auxin signaling pathway in plant species that diverged prior to the appearance of land plants is much less understood compared to what is known for land plants. Many studies have been reported that indole-3-acetic acid (IAA), which is the most common naturally occurring auxin in land plants, is actually produced by a broad range of photosynthetic organisms—from cyanobacteria to eukaryotic algae^22,23^. Indeed, exogenous application of IAA and other auxin compounds to those organisms affects their growth and other physiological processes. These findings raise the issue of whether a primitive auxin signaling pathway may have been established before the emergence of land plants.

In the last decade, the increase in the number of available genomic and transcriptomic sequences has promoted research on the presence of the nuclear auxin pathway in algal species. Both phylogenetic and structural analyses using those sequences suggested that several streptophyte algae, a paraphyletic group of land plants, partially possess ancestral versions of the components of the nuclear auxin pathway, but no dedicated TIR1/AFB receptor for auxin^13,24^. Thus, it is likely that a complete nuclear auxin pathway was established only in land plants, implying that a primitive auxin signaling pathway, i.e., not mediated by TIR1/AFB, functions in algal species. To date, however, the components of the auxin signaling pathway in algae have not been identified; importantly, identification of these components would help researchers elucidate of the evolution and molecular mechanism(s) underlying auxin signaling in algae.

We previously reported that IAA is produced by the streptophyte algae *Klebsormidium nitens*^25^, a non-branching, filamentous alga^26^, and that exogenous application of auxin suppresses cell proliferation in this alga^27^. Exogenously applied auxin also affects gene expression in *K. nitens*^13,27^, such as expression of the gene *Lateral Organ Boundary Domain 1* (*LBD1*), despite the fact that *K. nitens* lacks TIR1/AFB, Aux/IAA, and ARF^13,24,25^. Particularly, the expression of *LBD1* is induced within 1 h of auxin application, even in the presence of the translation inhibitor cycloheximide (CHX)^27^. These studies suggest that a TIR1/AFB-independent auxin signaling mechanism regulates gene expression and growth in *K. nitens*. Additionally, a homolog of the auxin transporter PIN-FORMED in *Klebsormidium flaccidum* localizes in the plasma membrane and facilitates auxin transport^28^, implying the existence of auxin-mediated cell-to-cell communication. Hence, information concerning the mechanism underlying the auxin response in *K. nitens* may provide insights into the primitive auxin signaling mechanism in algae and the evolution of auxin signaling in plants. At present, there are no established transformation procedures for *K. nitens*. However, gene expression profiling, introducing *K. nitens* genes into other organisms, and in vitro assays are useful analytical tools for research in *K. nitens*. Therefore, to identify the components of the auxin signaling pathway in *K. nitens,* we searched for cis-regulatory motifs and candidate proteins related to auxin-inducible gene expression and identified the *K. nitens* transcription factor RAV (KnRAV), which is associated with the auxin-induced transcriptional regulation in *K. nitens*.

## Results

### Exploration of the motif sequences related to the induction of gene expression by IAA in *K. nitens*

**Figure 1:**
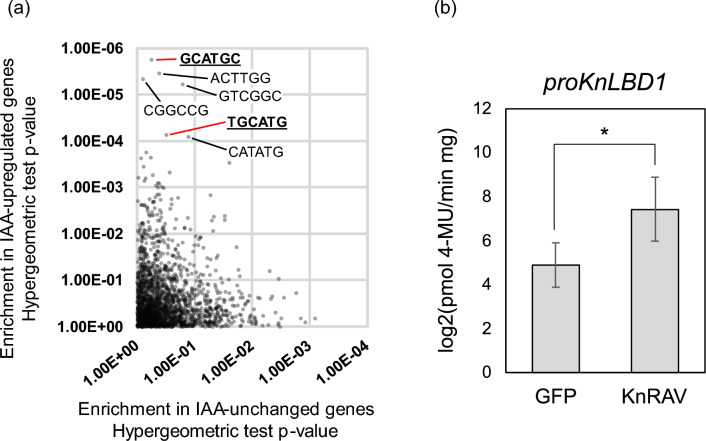
Exploration of a transcription factor related to IAA-inducible gene expression in *K. nitens*. (**a**) Comparison of the abundance of all 6-mer nucleotide sequences in the 1-kb region –900 to + 100 (relative to the transcription start site) between IAA-upregulated and IAA-non-responsive genes. The *P*-value for each 6-mer sequence was calculated with the hypergeometric test. (**b**) Quantification of GUS activity driven by the *KnLBD1* promoter in *N. benthamiana* leaves. Effector proteins were KnRAV and GFP (negative control). The *y* axis represents GUS activity on a log2 scale. Error bars represent SD of values for five replicates. *P < 0.05 (Student’s *t* test).

To predict which cis-regulatory elements and transcription factors are involved in the IAA response in *K. nitens*, we classified IAA-upregulated and unchanged genes using our previously reported RNA-sequencing data acquired for algae treated with IAA^27^, and then analyzed candidate motifs. A frequency analysis of all 2080 of the 6-mer nucleotide sequences comprised of reverse-complementary sequences revealed that certain 6-mer sequences were overly represented in promoters upstream of IAA-upregulated genes (Fig. 1a). Two of those sequences, 5′-TGCATG-3′ and 5′-GCATGC-3′ (Fig. 1a), matched the RY motif that constitutes a binding site for B3 domain transcription factors such as viviparous 1(VP1)/abscisic acid insensitive 3 (ABI3), FUSCA 3, and leafy cotyledon1^29–31^. The B3 domain transcription factors, found in chlorophytes and streptophytes^32^, compose a plant-specific superfamily. Throughout evolution, the B3 genes have undergone significant divergence, leading to the emergence of diverse transcription factors^33^, such as ARF and VP1/ABI3, involved in plant hormone signaling in land plants. Although the role of B3 genes in green algae is not well-understood, it is conceivable that these genes played a part in the development of the auxin signaling mechanism. Thus, we hypothesized that a B3 domain transcription factor that can bind the RY motif is involved in regulating gene expression in response to IAA in *K. nitens*. Based on a Pfam search^34^ of predicted proteins in *K. nitens*, nine B3 domain–containing proteins were identified, and the expression of eight of them was confirmed by RNA-seq (Supplemental Fig. 1).

### Identification of a transcription factor involved in regulating the expression of auxin-inducible genes in *K. nitens*

In search of a B3 domain transcription factor that could enhance the expression of IAA-upregulated genes, we utilized an agrobacterium infiltration–mediated transient expression system in *Nicotiana benthamiana* leaves for the simultaneous expression of the transcription factor candidate and β-glucuronidase (GUS) reporter constructs. The promoter sequence of *K. nitens LBD1* (*proKnLBD1*), which is likely to be directly regulated by auxin signaling in *K. nitens*^27^, was fused with the GUS reporter gene. Co-infiltration of leaves with one of the transcription factor candidates encoded by *kfl00094_0070* resulted in a clear increase in GUS activity driven by *proKnLBD1* (Supplemental Fig. 2), suggesting that *kfl00094_0070* might be involved in regulating *KnLBD1* expression. The gene *kfl00094_0070* encodes not only the B3 domain but also an AP2 DNA binding domain and a PB1 domain (Supplemental Fig. 1). In accordance with a previous report^13^, a phylogenetic analysis indicated that this transcription factor belongs to a group that contains the Related to ABI3/VP1 (RAV) transcription factor genes (Supplemental Fig. 3). Moreover, in contrast to our expectation, that previous report suggested that the B3 domain of KnRAV binds the 5′-CACCTG-3′ motif^24^ rather than the RY motif.

### KnRAV directly binds the promoter sequence of *KnLBD1* and enhances its expression

**Figure 2:**
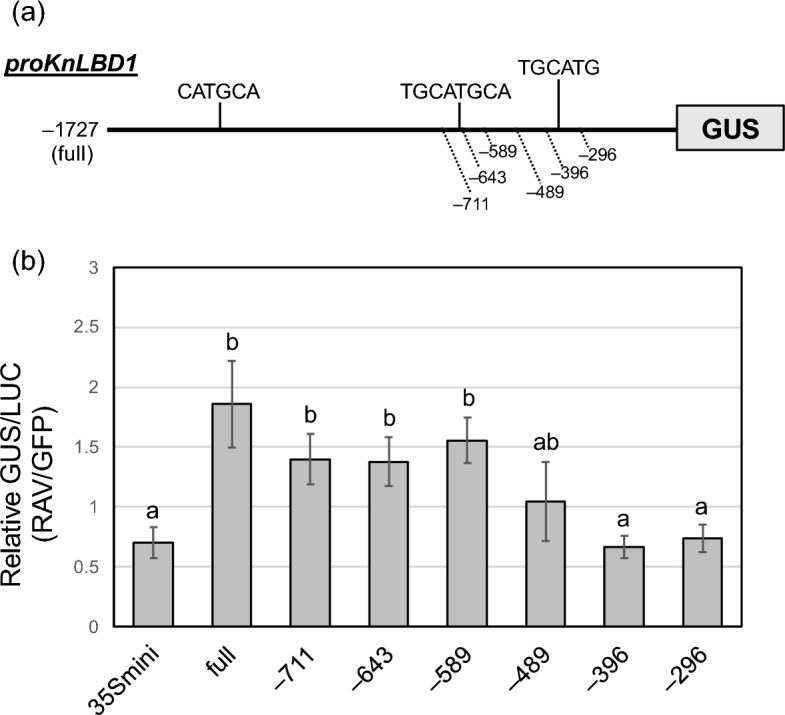
Identification of motifs responsible for DNA-binding of KnRAV. (**a**) Schematic representation of *proKnLBD1*. (**b**) Quantification of truncated *LBD1* promoter activity in *N. benthamiana* leaves. GUS activity driven by the *KnLBD1* promoter or 35S minimal promoter (pro35Smini) was normalized with *NOSp::LUC* activity. The *y* axis represents the ratio of GUS activity with KnRAV to that without KnRAV. Error bars represent SD of values for three replicates. Different lowercase letters denote a statistically significant difference between values. *P* < 0.05 (Tukey’s test).

To determine the sequence related to the regulation of gene expression by KnRAV, we examined the promoter activities of truncated *proKnLBD1* sequences (positions − 711, − 643, − 589, − 489, − 396 and − 296 relative to the transcription start site) (Fig. 2a) by calculating the ratio of *proKnLBD1*-fused GUS activity to nopaline synthase promoter (*pNOS*)-fused firefly luciferase (LUC) activity. Truncation of *proKnLBD1* at a position beyond − 489 from the start codon increased the GUS:LUC ratio upon co-expression with KnRAV, whereas the promoter up to − 489 did not significantly increase the GUS:LUC ratio compared with the negative control experiment with the cauliflower mosaic virus 35S minimal promoter (pro35Smini; Fig. 2b). Moreover, promoters with truncations at positions to − 396 and − 296 significantly decreased the responsiveness to KnRAV compared with promoters with truncations beyond − 489 (Fig. 2b). This suggested that sequences that are essential for KnRAV binding to the *KnLBD1* promoter exist in the region from − 589 to − 396 of *proKnLBD1*.

**Figure 3:**
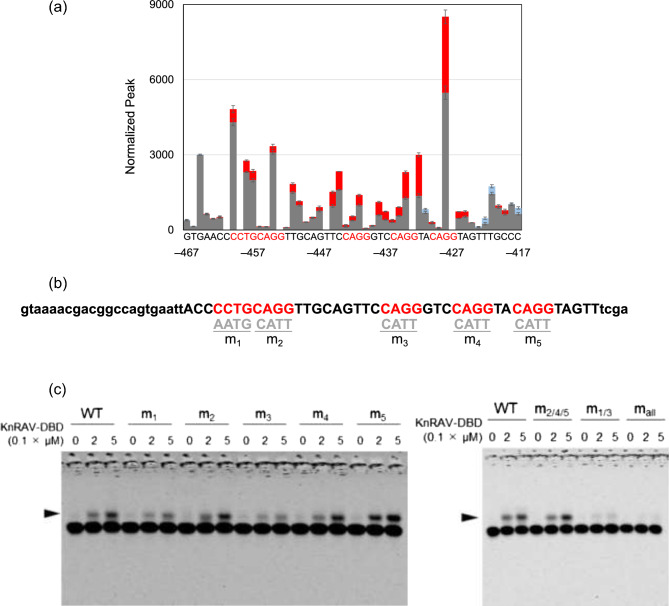
Identification of the nucleotide motifs responsible for DNA-binding of KnRAV. (**a**) DNase I footprint by the binding of His-tagged KnRAV-DBD to the FAM-labeled *KnLBD1* promoter fragment. This graph illustrates the peak values within the trimmed region (–467 to –417) of the graph shown in Supplementary 5c. Bar graph data represent mean values of normalized peak heights. The peak values lost in the presence of 3 µM KnRAV-DBD are represented by red coloring, and the peak values gained represented by blue coloring. The horizontal axis represents the corresponding position on *proKnLBD1*. Error bars represent SD of values for three replicates. (**b**) Sequences of the Cy5.5-labeled probes for EMSA. Red letters indicate 5′-CCTG-3′ and its reverse-complementary sequences. (**c** and **d**) Wild-type and mutated Cy5.5-labeled probes together with 0, 0.2, and 0.5 µM His-tagged KnRAV-DBD were used for EMSA. The arrowhead indicates the position of bands that were retarded in the presence of KnRAV-DBD. The electrophoresis image shows the range between sample wells and free fluorescent probe, including all bands in the gel.

Next, in vitro DNA binding assays were used to identify the exact sequence in *proKnLBD1* that was necessary for KnRAV binding. First, we expressed and purified a His-tagged recombinant protein, named KnRAV-DBD, comprising the AP2 region and the B3 DNA binding domain of KnRAV (Supplemental Fig. 4a,b). A DNase I footprinting assay using a sequence containing the − 589 to − 396 region of *proKnLBD1* revealed protection in the region with multiple 5′-CCTG-3′ sequences (Fig. 3a and Supplemental Fig. 5a–c). A previous study with *Arabidopsis thaliana* indicated that the B3 domain of *A. thaliana* RAV1 (AtRAV1) could preferentially bind the 5′-CACCTG-3′ motif but also could bind a broad range of target sequences containing 5′-CCTG-3′^35^. To test whether 5′-CCTG-3′ motifs contributed to KnRAV-DBD binding, 5′-Cy5.5-labeled DNA probes were prepared containing the sequence of the KnRAV-DBD-blocked region as determined by DNase I footprinting; mutants of 5′-CCTG-3′ were also prepared (Fig. 3b). An electrophoresis mobility shift assay (EMSA) demonstrated that co-incubation with KnRAV-DBD retarded the mobility of the fluorescently labeled probe with the wild-type sequence (WT; Fig. 3c, d). Among the five single-site mutants at each 5′-CCTG-3′, two showed a slight but clear reduction in the band shift (m_1_ and m_3_; Fig. 3c). In contrast, the remaining three did not (m_2_, m_4_, and m_5_; Fig. 3c). Both m_1_ and m_3_ mutations disrupt 5′-CCCTG-3′ sequences, while m_2_, m_4_ and m_5_ disrupt the 5’-ACCTG-3’ sequences, suggesting that the two 5′-CCCTG-3′ sites, corresponding to the m_1_ and m_3_ mutants, are important for the DNA binding of KnRAV-DBD. In addition, the m_5_ mutant probe obviously increased the intensity of the retarded band, implying that the mutation enhanced the binding between the adjacent 5′-CCTG-3′ sites and KnRAV-DBD. Next, we assessed the impacts of mutations at multiple 5′-CCTG-3′ sites on the DNA binding of KnRAV-DBD. The combination of m_1_ and m_3_ mutations (m_1/3_) strongly reduced the band shift compared to the WT probe, while the combination of m_2_, m_4_ and m_5_ mutations (m_2/4/5_) caused no significant changes (Fig. 3d). Furthermore, the nearly complete loss of the band shift was observed with the mutant probe in which all 5′-CCTG-3′ motifs were disrupted (m_all_). These results suggested that the KnRAV can preferentially bind to the adjacent 5′-CCCTG-3′ sites in the *KnLBD1* promoter and enhance its expression.

### KnRAV enhances the expression of multiple IAA-inducible genes

**Figure 4:**
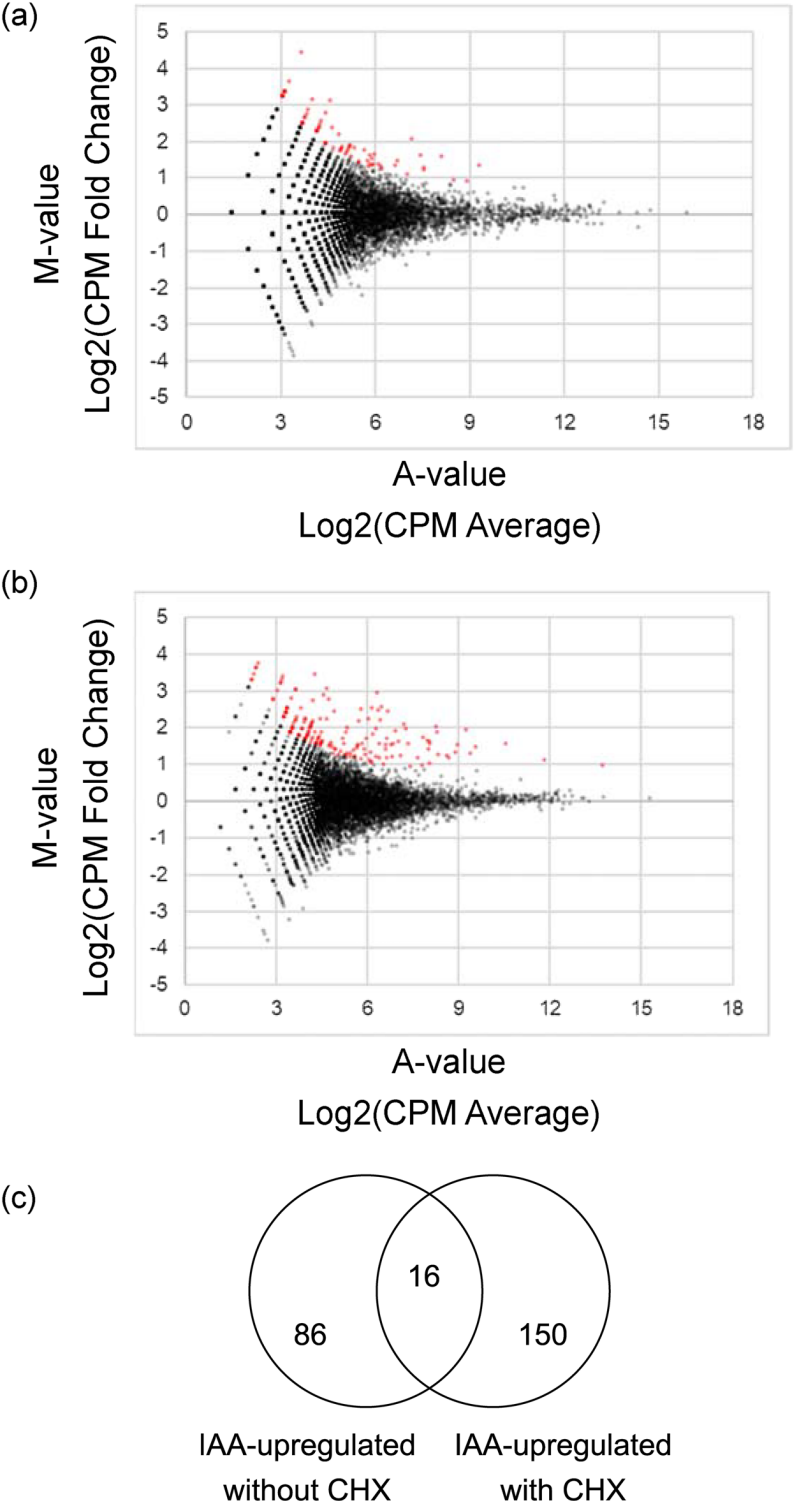
Transcriptome analysis of *K. nitens* using CAGE-seq. (**a** and **b**) MA plots were drawn with CAGE tag counts mapped to each cluster of CAGE tag start sites. This experiment was conducted with no biological replicate. The samples were treated with 0 or 100 µM IAA for 1 h in the absence (**a**) or presence of CHX (10 mg/L, b). Red dots represent differentially expressed clusters of CAGE tag start sites (*P* < 0.05). (**c**) Venn diagram representing subsets of IAA-upregulated genes in the absence or presence of CHX.

**Figure 5:**
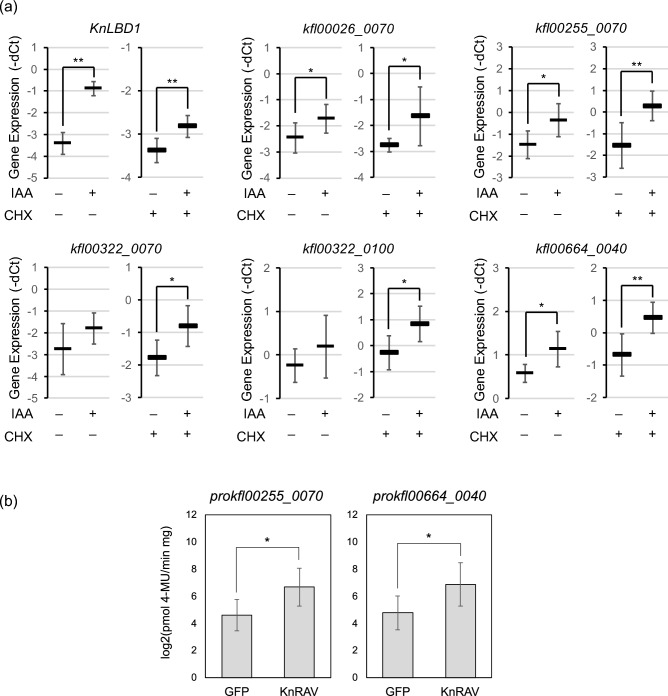
KnRAV can enhance the expression of multiple IAA-inducible genes in *K. nitens*. (**a**) Changes in the expression of IAA-upregulated genes classified with CAGE-seq data were analyzed by RT-qPCR. Samples were treated with 0 or 100 µM IAA in the absence or presence of CHX (10 mg/L) for 1 h. Error bars represent SD of values for six replicates. *P < 0.05; **P < 0.01 (Student’s *t* test). (**b**) Quantification of GUS activity driven by the *kfl00255_0070* or *kfl00664_0040* promoter in *N. benthamiana* leaves. Effector proteins were KnRAV and GFP (negative control). The *y* axis represents GUS activity on a log2 scale. Error bars represent SD of values for five replicates. *P < 0.05 (Student’s *t* test).

We evaluated whether KnRAV broadly regulates the expression of IAA-inducible genes. In our previous report, we demonstrated that treatment with IAA for more than 10 h resulted in substantial changes to the transcriptomes of *K. nitens*^27^. However, to identify genes that are directly regulated by the auxin response machinery in *K. nitens*, it is necessary to perform gene expression profiling on samples treated with IAA for shorter periods of time. Using *K. nitens* samples treated with IAA for 1 h, we carried out gene expression profiling for a period earlier than in our previous study^27^; Cap Analysis of Gene Expression (CAGE)-seq was used, which provides data for CAGE-tag read counts and CAGE-tag transcription start sites^36^. Moreover, some transcriptional regulators induced by IAA may alter the expression of genes not directly controlled by the auxin response machinery. Some of the IAA-treated samples were also treated with cycloheximide (CHX), an inhibitor of translation elongation during de novo protein synthesis^37^. This additional treatment aimed to further assess the expression of genes regulated by pre-existing transcriptional regulators early during the auxin response. Comparison of read counts mapped to the detected CAGE-tag transcription start-site clusters revealed that expression of a variety of genes was affected even at 1 h after treatment with exogenous IAA both in the absence and presence of CHX (Fig. 4a,b). IAA significantly increased the expression of 86 genes (no CHX) and 150 genes (with CHX) (Fig. 4c and Supplemental Data 1 and 2). In particular, 16 genes overlapped between the − CHX and + CHX groups (Fig. 4c and Supplemental Data 3). To confirm whether these genes could be reproducibly upregulated by IAA, we examined the expression of six genes, including *KnLBD1*, using reverse transcription and quantitative PCR (RT-qPCR). Four of these genes, namely *KnLBD1, kfl00026_0070*, *kfl00255_0070,* and *kfl00664_0040*, were reproducibly upregulated by IAA regardless of CHX (Fig. 5a). In contrast, *kfl00322_0070* and *kfl00322_0100* were significantly upregulated by IAA but only in the presence of CHX (Fig. 5a). Based on this gene expression analysis, we examined the ability of KnRAV to transactivate the *kfl00255_0070* and *kfl00664_0040* promoters. As with *proKnLBD1* (Fig. 1b), those two gene promoters were also significantly upregulated by KnRAV (Fig. 5b). These results indicated that KnRAV can enhance the expression of multiple genes upregulated by IAA, suggesting an association between auxin-induced gene expression and this transcription factor in *K. nitens*, where an auxin receptor TIR1/AFB is absent.

## Discussion

Auxin signaling, which is mediated by its receptor TIR1/AFB, is a ubiquitously conserved module that regulates gene expression in response to developmental cues and environmental stimuli. TIR1/AFB has been widely accepted as the key auxin receptor through studies with various land plants^13,16,18–20^. However, little is known about auxin signaling in algae, for which no dedicated TIR1/AFB homolog is found^13,24,25^.

To unravel the molecular mechanism of auxin signaling in algae, we explored transcription factors involved in auxin-inducible gene expression in *K. nitens*, which responds to auxin even without TIR1/AFB. We identified a transcription factor KnRAV that can enhance the expression of several IAA-inducible genes in *K. nitens* by cis-element analysis with transcriptome data and a promoter assay using *N. benthamiana* leaves (Fig. 1). Although the cis-element analysis suggested that the RY motif was enriched in the promoter region of IAA-inducible genes, in vitro DNA binding experiments demonstrated that KnRAV binds directly to 5′-CCTG-3′ motifs in *proKnLBD1* (Figs. 2 and 3), a binding motif for the B3 domain of AtRAV1^35^. These results suggest that KnRAV has the potential to regulate auxin-responsive gene expression in *K. nitens*. Our cis-element analysis with the promoters of auxin-responsive genes did not reveal significant enrichment of DNA sequences containing 5′-CCTG-3′ or 5′-CAACA-3′, a typical binding sequence for RAV-type AP2 domain (Fig. 1a). A Study on *A. thaliana* demonstrated that the recombinant AtRAV1 protein exhibits a preference for co-precipitating with DNA fragments that contain 5′-CAACA-3′ and 5′-CACCTG-3′ motifs in close proximity^35^. However, AtRAV1 has also been demonstrated to bind to a broad range of similar sequences as well. This is in line with the finding that, while KnRAV has a strong binding affinity for neighboring sequences of 5′-CAACA-3′ and 5′-CACCTG-3′^24^, it is also capable of binding to two adjacent 5′-CCCTG-3′ sequences in *KnLBD1* promoter (Fig. 3c,d). The diversity of binding sequences could make it challenging to reveal the relationship between KnRAV and auxin-inducible gene expression in *K. nitens* using the cis-element analysis. A more detailed characterization of the DNA binding mechanism of KnRAV could be necessary to address these challenges.

In contrast to our finding that KnRAV can activate *KnLBD1* (Fig. 1b), in *A. thaliana*, AtRAV1 acts as a transcriptional repressor that suppresses the expression of *AtABI3/4/5*, which encode transcription factors involved in ABA signaling^38^. It has been suggested that the repression mediated by AtRAV1 involves direct binding of the co-repressor TOPLESS (TPL) to the B3 repressor domain (BRD) located at the C-terminus of AtRAV1^39^. The BRDs (R/KLFGV sequence^40^) are highly conserved among plant RAV family proteins, including KnRAV^24^, implying the potential for KnRAV to bind to the conserved TPL^24^ and act as a transcriptional repressor in *K. nitens*. On the other hand, it has also been reported that AtRAV1 can activate the *Arabidopsis thaliana Oresara 1* gene in the presence of ABA^41^. Currently, the function of TOPLESS proteins in algae is not well understood. Investigating the functional interactions between KnRAV and TOPLESS, as well as the post-translational regulation of KnRAV, could be key to shedding light on the function of KnRAV.

Comparative genomic analyses suggest that the RAV and LBD genes were acquired after the emergence of streptophyte algae and are not present in chlorophytes^13,24,42^. While it is not yet clear if the induction of LBD genes by auxin is a common response in streptophyte algae, further investigation of the regulation of LBD gene expression by RAV transcription factors in other streptophyte algae could provide valuable insights.

In addition to KnRAV, RAV family genes in certain other streptophyte algae and in *M. polymorpha* also often encode not only B3 and AP2 DNA binding domains but also a PB1 domain^13,24^. The PB1 domain is commonly found in ARFs and AUX/IAAs and participates in multimer formation via interaction between PB1 domains in other ARFs and Aux/IAAs^11,12^. Those interactions play an important role in switching auxin-responsive gene expression in land plants. Although the PB1 domain of these RAV proteins is lacking in angiosperms, the relationship between the functions of those PB1-containing RAV proteins and the auxin response is worth investigating to reveal the evolutionary processes of auxin signaling in plants.

On the other hand, we found that the RY motif, i.e., 5′-TGCATG-3′, is clearly enriched in promoters of auxin-upregulated genes (Fig. 1), suggesting a relationship between the RY motif and IAA-inducible gene expression. In land plants, several B3 domain transcription factors, including VP1/ABI3, have been reported to bind the RY motif and control gene expression^29–31^. *proKnLBD1* encodes the typical RY motifs (Fig. 2a), but among the B3 domain–containing proteins we tested, only KnRAV strongly enhanced *proKnLBD1* expression*.* In addition, the in vitro DNA binding assay showed that the B3 domain of RAV does not bind the RY motif in *proKnLBD1* but rather 5′-CCTG-3′, a consensus motif for binding the B3 domain of AtRAV1^35^. Thus, the RY motif–binding protein remains to be identified. In angiosperms, the VP1/ABI3 proteins reportedly interact with other proteins to control the expression of specific genes^43^. For example, AtABI3 directly interacts with other proteins, such as the basic leucine zipper transcription factor abscisic acid insensitive 5^44^ and basic helix-loop-helix transcription factor phytochrome-interacting factor 1/phytochrome-interacting factor 3-like 5^45^, and coordinates with those proteins to modify gene expression. In *K. nitens*, gene expression that is activated by the binding of a transcription factor(s) to the RY motif perhaps requires multiple factors, including the putative RY motif–binding protein.

The available algal genome and transcriptome data indicate that B3 superfamily members have already been identified in some chlorophytes, but the number of B3 superfamily members has obviously increased during the evolution of streptophytes^17,33^. The increase in the number of the B3 genes, and the consequent divergent evolution, has led to the emergence of subfamilies with distinct functions^33^. Members of these subfamilies typically bind to different cis-element sequences. For instance, the VP1/ABI3 and its related transcription factors bind to the RY motif^29–31^, while ARFs bind to the auxin response element^46^. The expansion of B3 superfamily genes might constitute the molecular basis for the establishment of auxin signaling in plants during evolution.

Comparison of the gene expression analysis data using CAGE-seq and RT-qPCR demonstrated that a certain number of genes were upregulated even after 1 h of exogenous IAA treatment with or without CHX (Figs. 4 and 5). These data support our previous data suggesting that auxin signaling regulates gene expression in *K. nitens*^27^. Additionally, among the genes that were upregulated by IAA in the absence of CHX, most were not upregulated by IAA in the presence of CHX (Fig. 4). This implies that several transcriptional regulators other than KnRAV may be involved in the auxin response, constituting a signaling cascade through de novo protein synthesis. Since CHX might inhibit the translation of negative transcriptional regulators in response to auxin, many different genes may have been upregulated by IAA in the presence of CHX compared to its absence (Fig. 4).

It is still unclear whether the auxin response in algae is regulated through auxin-specific signaling machinery or is the consequence of the chemical properties of the auxin molecules^23^. In *K. nitens*, significant changes in gene expression, including the upregulation of *KnLBD1*, have been observed in response to treatment with exogenous IAA at a concentration of 100 µM, which is much higher than the typical endogenous levels^25,27^. To fully comprehend the physiological role of the observed response to excessive IAA application in *K. nitens*, it is necessary to identify components of the IAA response machinery. Moreover, a comprehensive understanding of the physiological role of IAA in *K. nitens* requires knowledge of the conditions under which IAA accumulates in this organism. Although our approach identified candidate transcription factors that may be involved in the initial response to auxin, the mechanism of auxin perception in *K. nitens* without the receptor TIR1/AFB remains unclear. However, the identification of upstream-acting factors, such as the putative protein that interacts with KnRAV, may provide important clues in this regard. Along with unraveling the mechanism of auxin signaling in *K. nitens*, evaluation how KnRAV contributes to the auxin response is also an important issue. This could be achieved via a genetic approach; until now, however, no transformation method has proved successful for *K. nitens*. Therefore, such an analysis will require the development of an appropriate technique for *K. nitens*. Recently, there has been an increase in the number of artificially transformable streptophyte algae such as *Closterium*^47,48^, *Penium*^49^, and *Mougeotia*^50^. Although it is not yet clear whether these algae have RAV transcription factors, if they do, characterization of RAV genes in these algae could help us better understand their contribution to auxin response in these algae.

Our results provide new insights into the regulation of IAA-inducible gene expression in *K. nitens*. Moreover, the findings offer not only knowledge about auxin signaling in this alga but also clues to understanding the evolution of auxin signaling in photosynthetic organisms.

## Materials and methods

### Plant materials

*N. benthamiana* was bred in our laboratory and approvals were not required for this study. *Klebsormidium nitens* NIES-2285 was provided by the National Institute for Environmental Studies (NIES Collection, Tsukuba, JAPAN). The collection, preservation, and use of plant materials involved in this study complied with relevant institutional, national, and international guidelines and legislation.

### RNA-seq analysis and cis-element analysis

Genes used for cis-element analysis were selected based on RNA-seq data at 10 h or 3 days of incubation of *K. nitens* samples in the absence or presence of 100 μM IAA as documented in a previous study (DDBJ Sequence Read Archive: DRR064061-064,064)^27^. RNA-seq reads were mapped to predicted mRNAs^25^ of *K. nitens* using HISAT2 (2.1.0)^51^. Read counts were normalized with iDEGES/edgeR using the TCC package (version 1.34.0)^52^. A total of 87 IAA-upregulated genes and 157 IAA-non-responsive genes were selected based on *P*-value cutoffs (upregulated: *P* < 0.01 at both 10 h and 3 days without IAA treatment; non-responsive: *P* > 0.9 at both 10 h and 3 days without IAA treatment, normalized read counts greater than 100). For all genes as well as the IAA-upregulated and IAA-non-responsive genes, 1-kbp sequences located − 900 to + 100 upstream of the gene loci were extracted. The presence or absence of all 2080 of the 6-mer nucleotide sequences comprised of reverse-complimentary sequences was determined using custom Perl scripts. For each 6-mer sequence, the *P*-values for the frequency in both the IAA-upregulated and IAA-non-responsive groups were calculated with the hypergeometric test against the frequency in all genes.

### Plasmid construction

Primers listed in Supplemental Data 4 were used for plasmid construction. For GUS assay constructs, gene promoters were inserted upstream of *GUS* in vector pBI121 using the in vivo* E. coli* cloning (iVEC) method^53^. The pBI121 sequence was amplified by PCR with primers pBI121_right_R and GUS_F. For homologous recombination, the promoter sequences were amplified with primers containing the terminal sequences of the fragment from pBI121 in those 5’-ends. Genomic DNA of *K. nitens* was used as the PCR template. Those pBI121 and gene promoter fragments were fused via transformation of *E. coli* strain iVEC3^53^.

For the *GUS* and *LUC* constructs, DNA fragments were fused by homologous recombination using the SLiCE method^54^. First, the 35S promoter region was removed by self-recombination of PCR products amplified with primers GUS_F_xhoI_pBI121_self and NOSter_R_xhoI_pBI121_self. The *GUS* cassette was amplified from pBI121 and the 35S promoter was deleted via PCR with primers M13_Fwd and pBI121_NOSter_pEGB3_Fluc_fusion_F. Next, the *GUS* cassette was introduced to the pEGB3 backbone that already had been amplified with primers Rluc_Cter_F and Fluc_Cter_R. Finally, the 70-bp fragment by primer extension with primers pBI121_self_pvuII-XhoI_F and pBI121_self_pvuII-XhoI_R was inserted into the aforementioned modified plasmid pEGB3 between the PvuII and XhoI sites. This plasmid, pLG_XhoI, was digested with XhoI, and then the truncated pro*KnLBD1* sequences were inserted into the plasmid.

To identify candidates for B3 domain transcription factors, a domain search was performed using amino acid sequences for all *K. nitens* proteins using the Pfam v27.0 database^34^.

As the expression vector for those B3 domain containing proteins, pBI121-XS was constructed via XhoI digestion and self-ligation of PCR products amplified with primers ivec_pBI121_XhoI_fwd and ivec_pBI121_XhoI_rev. Next, the coding sequences of the predicted B3 domain–containing genes were amplified from *K. nitens* cDNA with primers containing the recombinant sites and fused to the PBI121-XS backbone amplified with primers ivec_35S_R and pBI121_left_F using the iVEC method.

For expression of recombinant KnRAV in *E. coli*, the sequence containing AP2 and the B3 DNA binding domain of KnRAV was amplified by PCR with primers EcoRI_NdeI_KnRAV_DBD_F and SalI_KnRAV_DBD_R. This fragment and pET-21b( +) plasmid were digested with NdeI and SalI and ligated into pET21b( +) to yield construct pET21b(+)/KnRAV-DBD.

For preparation of 6-Carboxyfluorescein (FAM)-labeled probes, the *proKnLBD1* sequence was amplified from the GUS assay construct with primers EcoRI_KnLBD1_pro_-589_F and HindIII_KnLBD1_pro_-296_R. This fragment and plasmid pUC19 were digested with EcoRI and HindIII and fused by ligation.

For EMSA, DNA fragments (from position –462 to –421) and mutated sequences encoded in *proKnLBD1* were prepared by annealing pairs of DNA oligos. The annealed fragments were ligated into pUC19 between the EcoRI and SalI sites.

### Agroinfiltration of *N. benthamiana* leaves

For calculation of GUS activity and the GUS:LUC ratio, agroinfiltration was performed with *Agrobacterium tumefaciens* strain GV3101(pMP90). The *A. tumefaciens* cells were transformed with the *GUS* and *GUS* + *LUC* plasmids and grown in liquid YEP medium at 30 °C for 20 h. Prior to agroinfiltration, *N. benthamiana* plants were grown for 5 weeks under continuous light (40–50 μmol m^−2^ s^−1^). The culture and infiltration of *A. tumefaciens* was carried out as described previously^55^. After 2 days of infiltration, 5 mm squares of the infected leaves were excised and collected into 1.5 mL tubes and frozen in liquid nitrogen.

### GUS assay

For quantification of GUS activity, frozen samples of *N. benthamiana* leaf were ground in 100 µL GUS extraction buffer (50 mM sodium phosphate pH 7.0, 10 mM 2-mercaptoethanol, 10 mM Na_2_EDTA, 0.1% sodium lauryl sarcosine, 0.1% (v/v) Triton X-100). After centrifugation at 13,000×*g* at 4 °C for 5 min, the supernatant was collected in another tube. This supernatant (50 µL) was mixed with an equal volume of GUS assay solution (2 mM 4-methylumbelliferyl-d-glucuronide [4-MU] in the extraction buffer) and incubated at 37 °C. For each time point (0, 30, and 60 min), a 20 µL aliquot was collected and the reaction stopped by adding 980 µL of 0.2 M sodium carbonate. The fluorescence intensity of 4-MU was quantified using a multi-label plate reader (EnSpire 2300-00J, Perkin-Elmer). Additionally, 10 µL of the supernatant was used for protein quantification with the Bradford reagent.

### Measurement of the GUS:LUC ratio

The GUS:LUC ratio was calculated as described previously^56^. The frozen samples of *N. benthamiana* leaf ground in 200 µL grinding buffer (100 mM potassium phosphate pH 7.8, 1 mM EDTA, 7 mM 2-mercaptoethanol, 0.1% (v/v) Triton X-100, 10% (v/v) glycerol). After centrifugation at 13,000×*g* at 4 °C for 5 min, 40 µL of the supernatant was collected in another tube and diluted fourfold with grinding buffer. For quantification of GUS activity, 50 µL of each diluted sample was mixed with 50 µL of GUS assay solution (2 mM of 4-MU in grinding buffer), and fluorescence was quantified as described above.

To quantify LUC activity, 20 µL of each aforementioned diluted sample was further diluted fivefold with grinding buffer. Then, 10 µL of that diluted sample was used to quantify LUC fluorescence using a PIKAGENA assay kit (Toyo Ink).

### Purification of recombinant KnRAV-DBD

*Escherichia coli* ArcticExpress (DE3) RIL (Agilent Technologies) transfected with pET21b(+)/KnRAV-DBD was pre-cultured in 20 mL of LB medium containing 100 mg/L ampicillin, 20 mg/L gentamicin, and 1% glucose at 37 °C overnight with shaking. Then, 10 mL of the overnight culture was each inoculated into two 500 mL volumes of LB medium without antibiotics and cultured at 30 °C with shaking. After growing to OD_600_ = 0.5, the culture medium was transferred to 13 °C and incubated overnight in the presence of 0.4 mM isopropyl-β-d-thiogalactopyranoside. The cells were collected by centrifugation at 2000×*g* at 4 °C for 10 min and suspended in 12.5 mL sonication buffer (20 mM Tris-HCl pH 8.0; 300 mM NaCl; 1 mM dithiothreitol), and the cell suspension was disrupted with an ULTRASONIC DISRUPTOR UD-211 (TOMY) and centrifuged at 20,000×*g* at 4 °C for 5 min. The supernatant was collected in another tube and used for His-tag purification with His60 Ni Superflow (Clontech) and a TALON 2 mL Disposable Gravity Column (TaKaRa) in accordance with the instruction manual. To remove the imidazole-containing elution buffer, the column eluent was dialyzed in 500 mL storage buffer (20 mM Tris-HCl pH 8.0, 300 mM NaCl, 1 mM EDTA, 1 mM dithiothreitol). During dialysis overnight at 4 °C, the buffer was replaced twice. The dialyzed content was divided into small aliquots and stored at − 80 °C until use.

### DNase I footprinting

The FAM-labeled DNA probe was prepared for DNase I footprinting assay. Plasmid pUC19 containing the *proKnLBD1* sequence from − 589 to − 296 was amplified by PCR with primers FAM_M13_Fwd and KnLBD1_excised_-296_EMSA and extracted using the Wizard SV Gel and PCR Clean-Up System (Promega). Then, 100 fmol of FAM-labeled probe was mixed with 50 µL of 2 × buffer (20 mM Tris-HCl pH 7.5, 100 mM KCl, 10 mM MgCl_2_, 0.2 mM CaCl_2_), 2 µg salmon sperm DNA, and 2 µL of 5 mg/mL bovine serum albumin (dissolved in the aforementioned storage buffer) and brought to 90 µL with nuclease-free water. The pre-diluted His-tagged KnRAV-DBD (5 µL) was added to the reaction mixture to a final concentration of 3 μM with subsequent incubation at 25 °C for 30 min. Prior to DNase I digestion, recombinant DNase I (TaKaRa) was diluted to 10 mM with its storage buffer (20 mM Tris-HCl pH 7.5, 50 mM NaCl, 0.1 mM CaCl_2_, 50% (v/v) glycerol). The diluted DNase I solution (5 µL) was added to the reaction mixture with incubation at 25 °C for 2 min. To stop the reaction, phenol–chloroform extraction was performed with an equal volume of acid phenol:chloroform (1:1). After centrifugation at 20,000×*g* at 4 °C for 5 min, the supernatant was ethanol precipitated and rinsed twice with 70% ethanol. The pellet was dissolved in 15 μL HiDi formamide (Thermo Fisher Scientific) that contains GeneScan 600 LIZ dye Size Standard (Thermo Fisher Scientific). The DNase I–digested DNA fragments were detected using a 3730xl DNA Analyzer (Thermo Fisher Scientific) and analyzed with Peak Scanner 2 (Applied Biosystems). The corresponding position in *proKnLBD1* was predicted based on the electropherogram of the PCR products amplified with primer FAM_M13_Fwd and reverse primers specific for sequences starting at positions − 296, − 368, − 403, − 442, − 476, and − 532. For comparative analysis of electropherograms between samples, the detected peaks were normalized. For each peak, the log2 ratio of the peak mean of 0 μM KnRAV-DBD in three samples to that of 3 μM KnRAV-DBD in three samples was calculated, and the lowest 30% and the highest 30% peaks were discarded. Using the remaining peaks, normalized factors were calculated to yield a zero-sum of the log2 ratios of the peak of each sample to the mean peak of the 0 μM KnRAV-DBD in three samples.

### EMSA

The sequences of the plasmids created for EMSA were amplified with primers Cy5.5_M13_Fwd and SalI_pLBD1_-462to-421_R or mutant primers. The PCR products were purified with the MinElute Gel Extraction kit (Qiagen). Cy5.5-labeled probes (100 fmol) were each mixed with 10 µL of 2 × binding buffer (20 mM Tris-HCl pH 7.5, 0.2% (v/v) Triton X-100, 5% (v/v) glycerol), 2 µL of 1 mg/mL BSA, 2 µL of 1 M KCl, and 20 µg/mL heparin and brought to 19 µL with nuclease-free water. The pre-diluted His-tagged KnRAV-DBD protein (1 µL) was added to the mixture to a final concentration of 0, 0.2, or 0.5 μM, and with incubation at 25 °C for 30 min. After pre-running the gel (2% agarose) for 60 min at 50 V, the samples were subjected to electrophoresis using 0.25 × TBE buffer for 30 min at 100 V. The entire electrophoresis procedure was carried out at room temperature. The fluorescence of Cy5.5-labeled probes was detected with Odyssey Classic (RI-COR). The overall images of the EMSA electrophoresis are shown in Supplementary Fig. 6.

### Culture conditions

*K. nitens* NIES-2285 was subcultured in 50 mL BCDAT liquid medium containing 0.1% glucose with aeration at 23 °C under 10 to 20 μmol photons m^−2^ s^−1^ of light for 2 weeks, referring to our previous study^27^.

For RNA samples, the cells were concentrated via centrifugation at 130×*g* for 10 min (LC-1000 7050-02 swinging-bucket rotor, TOMY, Tokyo, Japan). The cell pellet was transferred to a 15 mL centrifuge tube, resuspended in fresh medium, and centrifuged at 130×*g* for 5 min; the supernatant was discarded. Each pellet was washed three times with fresh medium and then resuspended with fresh medium (equivalent to eightfold of the packed-cell volume). For CAGE-seq, 500 mL fresh medium was inoculated with 10 mL of the suspension, and, for RT-qPCR, 50 mL fresh medium was inoculated with 1 mL of the suspension, followed by 24 h of pre-culture. The resulting cultured cells were left untreated or treated with IAA (final concentration, 100 µM) for 1 h in the absence or presence of CHX (final concentration, 10 mg/L). IAA was dissolved in 1 N NaOH for a 500 mM stock solution, and CHX was dissolved in dimethyl sulfoxide for a 10 mg/mL stock solution. Finally, the samples were collected on a nitrocellulose membrane by vacuum filtration and frozen in liquid nitrogen.

### RNA preparation

Total RNA was extracted from *K. nitens* referring to our previous study^27^. The aforementioned frozen *K. nitens* samples were ground into a fine powder with a mortar and pestle. At least three volumes of RNA extraction buffer (0.8% SDS, 25 mM Tris-HCl pH 7.6, 25 mM MgCl_2_, 25 mM KCl) and acid phenol were each added to the sample. After further grinding, the samples were collected into 2 mL tubes and centrifuged at 20,000×*g* at 4 °C for 10 min. The aqueous phase was liquid–liquid extracted three times with an equal volume of acid phenol:chloroform (1:1). The extracted aqueous samples were subjected to isopropanol precipitation and rinsed with 70% ethanol. The pellets were dissolved in RNase-free water, treated with recombinant DNase I (TaKaRa) in accordance with the user manual, extracted twice with acid phenol:chloroform (1:1), and precipitated with ethanol. Each pellet was dissolved in RNase-free water and used for RT-qPCR.

For CAGE-seq, the RNA samples were mixed with one-third volume of 10 M lithium chloride and incubated for at least 1 h at − 20 °C. After centrifugation at 20,000×*g* at 4 °C for 10 min, each pellet was washed with a solution containing 2 M lithium chloride and 50 mM EDTA and rinsed with 70% ethanol. For further purification, the RNeasy Mini or Midi kit (Cat. No. 74104/75142, Qiagen) was used before CAGE-seq.

### CAGE-seq analysis

The CAGE library was prepared and subjected to sequencing by DNAFORM (Yokohama, Kanagawa, Japan). CAGE tags were mapped to the *K. nitens* genome (version 1.0)^25^ using HISAT2 (version 2.2.1)^51^ after masking ribosomal RNA. For discarding ribosomal RNA sequences, we referred to the general feature format (GFF) file (version 1.1)^25^ and the results of infernal (version 1.1.4)^57^. CAGEr (version 2.2.0)^58^ was used for detection and clustering of CAGE-tag start sites. Normalization via trimmed mean of M-values and statistical analysis of the clusters were performed with edgeR (version 3.38.1)^59^.

### RT-qPCR

cDNA was synthesized with SuperScript III Reverse Transcriptase (Invitrogen) and oligo(dT). qPCR was performed with TB Green Premix Ex Taq II (Tli RNase H Plus) (TaKaRa) and the Thermal Cycler Dice Real Time System III following the instructions of the manufacturer. The citrate synthase gene, kfl00009_0420, was used for the housekeeping gene. Supplementary Data 4 lists the primers for RT-qPCR.

## Supplementary Information

Supplementary Figures.

Supplementary Information 1.

## Data Availability

The data that support the findings of our study are available from the corresponding author upon reasonable request. Raw read data for CAGE-seq can be accessed in the DDBJ Sequence Read Archive under accession numbers DRR428547-DRR428550. All experiments were performed in accordance with relevant guidelines and regulations.

## References

[1] Santner A, Calderon-Villalobos LI, Estelle M (2009). Plant hormones are versatile chemical regulators of plant growth. Nat. Chem. Biol..

[2] Wolters H, Jürgens G (2009). Survival of the flexible: Hormonal growth control and adaptation in plant development. Nat. Rev. Genet..

[3] Waadt R (2022). Plant hormone regulation of abiotic stress responses. Nat. Rev. Mol. Cell. Biol..

[4] Friml J (2003). Efflux-dependent auxin gradients establish the apical-basal axis of Arabidopsis. Nature.

[5] Stepanova AN (2008). TAA1-mediated auxin biosynthesis is essential for hormone crosstalk and plant development. Cell.

[6] Yamada M, Greenham K, Prigge MJ, Jensen PJ, Estelle M (2009). The TRANSPORT INHIBITOR RESPONSE2 gene is required for auxin synthesis and diverse aspects of plant development. Plant Physiol..

[7] Berleth T, Mattsson J, Hardtke CS (2000). Vascular continuity and auxin signals. Trends Plant Sci..

[8] Friml J, Wiśniewska J, Benková E, Mendgen K, Palme K (2002). Lateral relocation of auxin efflux regulator PIN3 mediates tropism in Arabidopsis. Nature.

[9] Okushima Y (2005). Functional genomic analysis of the AUXIN RESPONSE FACTOR gene family members in Arabidopsis thaliana: unique and overlapping functions of ARF7 and ARF19. Plant Cell.

[10] Möller B, Schenck D, Lüthen H (2010). Exploring the link between auxin receptors, rapid cell elongation and organ tropisms. Plant Signal. Behav..

[11] Lavy M, Estelle M (2016). Mechanisms of auxin signaling. Development.

[12] Salehin M, Bagchi R, Estelle M (2015). SCFTIR1/AFB-based auxin perception: Mechanism and role in plant growth and development. Plant Cell.

[13] Mutte SK (2018). Origin and evolution of the nuclear auxin response system. Elife.

[14] Rensing SA (2008). The *Physcomitrella* genome reveals evolutionary insights into the conquest of land by plants. Science.

[15] Lang D (2018). The *Physcomitrella* patens chromosome-scale assembly reveals moss genome structure and evolution. Plant J..

[16] Lavy M (2016). Constitutive auxin response in *Physcomitrella* reveals complex interactions between Aux/IAA and ARF proteins. Elife.

[17] Bowman JL (2017). Insights into land plant evolution garnered from the *Marchantia polymorpha* genome. Cell.

[18] Flores-Sandoval E, Eklund DM, Bowman JL (2015). A simple auxin transcriptional response system regulates multiple morphogenetic processes in the liverwort *Marchantia polymorpha*. PLoS Genet..

[19] Kato H (2015). Auxin-mediated transcriptional system with a minimal set of components is critical for morphogenesis through the life cycle in *Marchantia polymorpha*. PLoS Genet..

[20] Kato H (2020). Design principles of a minimal auxin response system. Nat. Plants.

[21] Li FW (2020). Anthoceros genomes illuminate the origin of land plants and the unique biology of hornworts. Nat. Plants.

[22] Tan CY (2021). Regulation of algal and cyanobacterial auxin production, physiology, and application in agriculture: An overview. J. Appl. Phycol..

[23] Lau S, Shao N, Bock R, Jürgens G, De Smet I (2009). Auxin signaling in algal lineages: Fact or myth?. Trends Plant Sci..

[24] Martin-Arevalillo R (2019). Evolution of the Auxin Response Factors from charophyte ancestors. PLoS Genet..

[25] Hori K (2014). Klebsormidium flaccidum genome reveals primary factors for plant terrestrial adaptation. Nat. Commun..

[26] Rindi F, Mikhailyuk TI, Sluiman HJ, Friedl T, López-Bautista JM (2011). Phylogenetic relationships in Interfilum and Klebsormidium (Klebsormidiophyceae, Streptophyta). Mol. Phylogenet. Evol..

[27] Ohtaka K, Hori K, Kanno Y, Seo M, Ohta H (2017). Primitive auxin response without TIR1 and Aux/IAA in the charophyte alga *Klebsormidium nitens*. Plant Physiol..

[28] Skokan R (2019). PIN-driven auxin transport emerged early in streptophyte evolution. Nat. Plants.

[29] Suzuki M, Kao CY, McCarty DR (1997). The conserved B3 domain of VIVIPAROUS1 has a cooperative DNA binding activity. Plant Cell.

[30] Kroj T, Savino G, Valon C, Giraudat J, Parcy F (2003). Regulation of storage protein gene expression in Arabidopsis. Development.

[31] Mönke G (2004). Seed-specific transcription factors ABI3 and FUS3: Molecular interaction with DNA. Planta.

[32] Carbonero P, Iglesias-Fernández R, Vicente-Carbajosa J (2017). The AFL subfamily of B3 transcription factors: Evolution and function in angiosperm seeds. J. Exp. Bot..

[33] Romanel EA, Schrago CG, Couñago RM, Russo CA, Alves-Ferreira M (2009). Evolution of the B3 DNA binding superfamily: New insights into REM family gene diversification. PLoS ONE.

[34] Finn RD (2014). Pfam: The protein families database. Nucleic Acids Res..

[35] Kagaya Y, Ohmiya K, Hattori T (1999). RAV1, a novel DNA-binding protein, binds to bipartite recognition sequence through two distinct DNA-binding domains uniquely found in higher plants. Nucleic Acids Res..

[36] Murata M (2014). Detecting expressed genes using CAGE. Methods Mol. Biol..

[37] de Garreau LN (2014). Structural basis for the inhibition of the eukaryotic ribosome. Nature.

[38] Feng CZ (2014). Arabidopsis RAV1 transcription factor, phosphorylated by SnRK2 kinases, regulates the expression of ABI3, ABI4, and ABI5 during seed germination and early seedling development. Plant J..

[39] Collins J, O'Grady K, Chen S, Gurley W (2019). The C-terminal WD40 repeats on the TOPLESS co-repressor function as a protein-protein interaction surface. Plant Mol Biol..

[40] Ikeda M, Ohme-Takagi M (2009). A novel group of transcriptional repressors in Arabidopsis. Plant Cell Physiol..

[41] Zhao Y (2016). ABA receptor PYL9 promotes drought resistance and leaf senescence. Proc. Natl. Acad. Sci. U. S. A..

[42] Chanderbali AS, He F, Soltis PS, Soltis DE (2015). Out of the water: Origin and diversification of the LBD gene family. Mol. Biol. Evol..

[43] Bulgakov VP, Koren OG (2022). Basic protein modules combining abscisic acid and light signaling in *Arabidopsis*. Front. Plant Sci..

[44] Nakamura S, Lynch TJ, Finkelstein RR (2001). Physical interactions between ABA response loci of *Arabidopsis*. Plant J..

[45] Park J, Lee N, Kim W, Lim S, Choi G (2011). ABI3 and PIL5 collaboratively activate the expression of SOMNUS by directly binding to its promoter in imbibed *Arabidopsis* seeds. Plant Cell.

[46] Ulmasov T, Hagen G, Guilfoyle TJ (1999). Activation and repression of transcription by auxin-response factors. Proc. Natl. Acad. Sci. U. S. A..

[47] Abe J (2011). Stable nuclear transformation of the *Closterium peracerosum-strigosum-littorale* complex. Plant Cell Physiol..

[48] Kawai J (2022). Highly efficient transformation of the model zygnematophycean alga *Closterium peracerosum-strigosum-littorale* complex by square-pulse electroporation. New phytol..

[49] Sørensen I (2014). Stable transformation and reverse genetic analysis of Penium margaritaceum: A platform for studies of charophyte green algae, the immediate ancestors of land plants. Plant J..

[50] Regensdorff M (2018). Transient genetic transformation of *Mougeotia scalaris* (Zygnematophyceae) mediated by the endogenous α-tubulin1 promoter. J Phycol..

[51] Kim D, Paggi JM, Park C, Bennett C, Salzberg SL (2019). Graph-based genome alignment and genotyping with HISAT2 and HISAT-genotype. Nat. Biotechnol..

[52] Sun J, Nishiyama T, Shimizu K, Kadota K (2013). TCC: An R package for comparing tag count data with robust normalization strategies. BMC Bioinform..

[53] Nozaki S, Niki H (2019). Exonuclease III (XthA) enforces In Vivo DNA cloning of *Escherichia coli* to create cohesive ends. J. Bacteriol..

[54] Okegawa Y, Motohashi K (2015). A simple and ultra-low cost homemade seamless ligation cloning extract (SLiCE) as an alternative to a commercially available seamless DNA cloning kit. Biochem. Biophys. Rep..

[55] Ma S, Dinesh-Kumar S (2014). SGR-based reporter to assay plant transcription factor-promoter interactions. Bio Protoc..

[56] Kanchiswamy C (2010). Regulation of Arabidopsis defense responses against *Spodoptera littoralis* by CPK-mediated calcium signaling. BMC Plant Biol..

[57] Nawrocki EP, Eddy SR (2013). Infernal 1.1: 100-fold faster RNA homology searches. Bioinformatics.

[58] Haberle V, Forrest AR, Hayashizaki Y, Carninci P, Lenhard B (2015). CAGEr: precise TSS data retrieval and high-resolution promoterome mining for integrative analyses. Nucleic Acids Res..

[59] Robinson MD, McCarthy DJ, Smyth GK (2010). edgeR: A Bioconductor package for differential expression analysis of digital gene expression data. Bioinformatics.

