# Supplementary material for: Cis-regulatory elements and transcription factors related to auxin signaling in the streptophyte algae Klebsormidium nitens
Source: Sci Rep. 2023 Jun 15;13:9635. doi: 10.1038/s41598-023-36500-x (PMC10272232; doi:10.1038/s41598-023-36500-x)
Supplement: Supplementary file 1 — Supplementary Figures. [file 41598_2023_36500_MOESM1_ESM.docx]

**
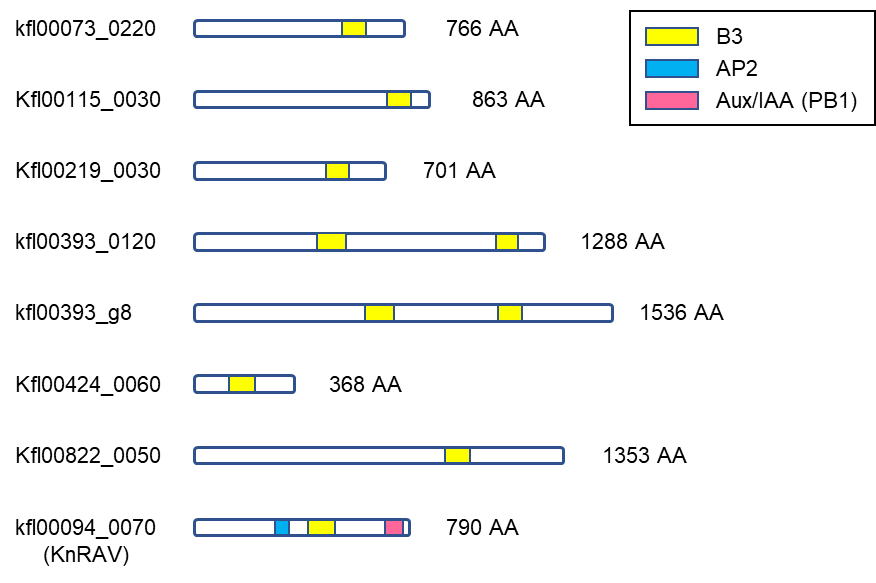
**

**Supplementary Figure 1. B3 domain transcription factor candidates in *K. nitens*.**

Schematic representation of the B3 domain transcription factor candidates identified with Pfam database (version27.0). Domains’ above the current color key shown in the figure.


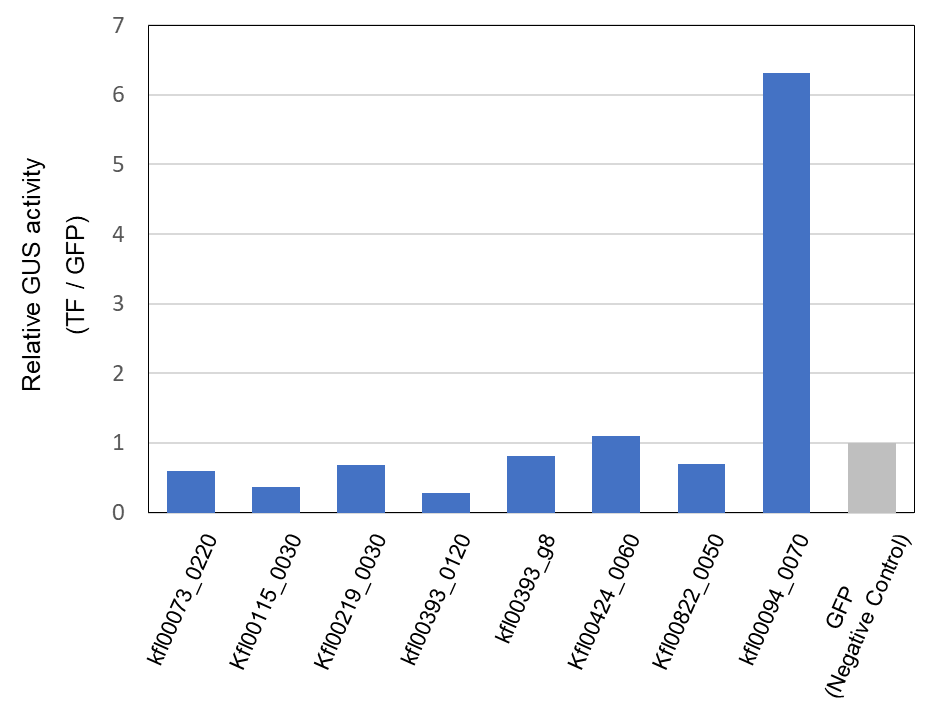


**Supplementary Figure 2. Exploration of a transcription factor that could transactivate the IAA-inducible gene *KnLBD1***

Quantification of GUS activity driven by the *KnLBD1* promoter in *N. benthamiana* leaves. Effector proteins, the B3 transcription factors, and GFP (negative control) were simultaneously expressed. Bar graphs represent GUS activity normalized to the value measured for the GFP control. This experiment was conducted with no biological replicate.

**
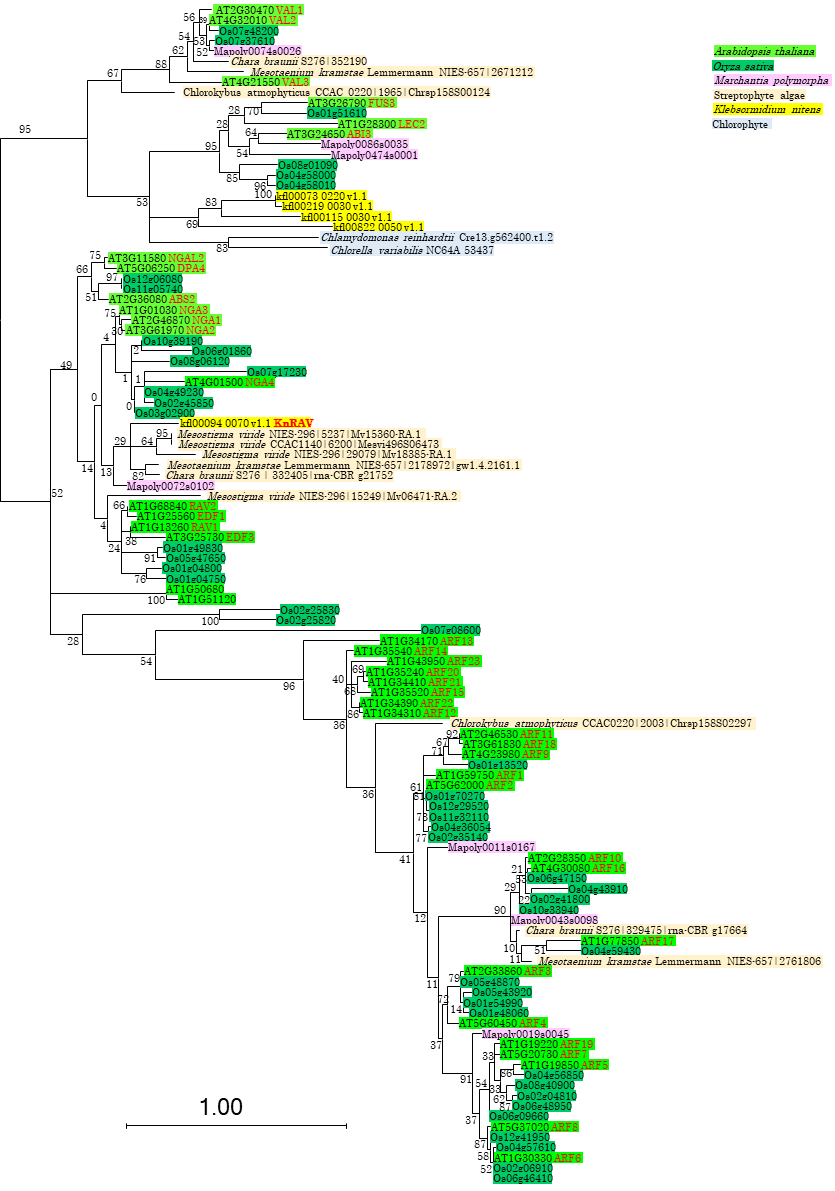
**

**Supplementary Figure 3. Phylogenetic analysis with B3 domain proteins in plants.**

B3 domain sequences of *Arabidopsis thaliana*, *Oryza sativa*, *Marchantia polymorpha*, *Klebsormidium nitens*, streptophyte algae, and chlorophyte were obtained by BLASTP search from Phytozome and Phycocosm database (query: KnRAV; E-value: <1e-5). Phylogenetic analysis using maximum likelihood was performed in MEGAX (the ML heuristic method: the Subtree–Pruning–Regrafting algorithm with search level 3; a branch swap filter: Moderate; the amino acid substitution model: the LG + F with 8 gamma categories and invariant sites). Bootstrap values represent data for 500 replicates. The tree was drawn to scale, with each branch length indicating the number of substitutions per site.

**(a)**

**PB1**

**B3**

**AP2**

**KnRAV**

**AP2**

**B3**

**KnRAV-DBD (6xHis)**

**-6xHis**

**
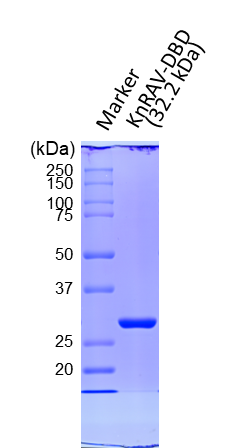
(b)**

**Supplementary Figure 4. Purification of His-tagged KnRAV-DBD.**

(a) Schematic representation of the cloned domain region of KnRAV for the in vitro DNA binding assay. Yellow, blue, and red coloring represents the predicted positions of the B3, AP2, and PB1 domains, respectively. (b) Coomassie blue staining of an SDS-PAGE gel of recombinant KnRAV-DBD (arrowhead) purified with Ni-IDA resin.


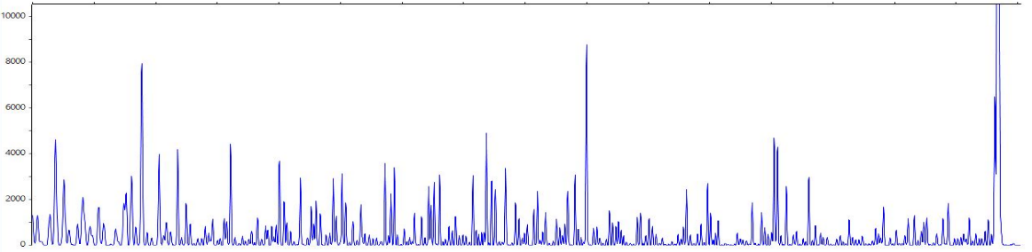
(a) 0 µM KnRAV-DBD


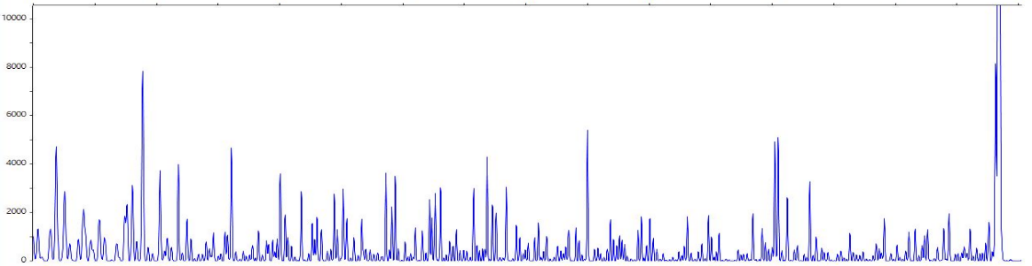
(b) 3 µM KnRAV-DBD

(c)

: 0 µM KnRAV-DBD

: 3 µM KnRAV-DBD

Normalized peak height

**Supplementary Figure 5. DNase I footprinting with the KnRAV-DBD protein and *proKnLBD1* sequence.**

(a and b) Electropherogram of peaks resulting from digestion with DNase I in the absence (0 µM, a) or presence of KnRAV-DBD (3 µM, b). (c) Mean values for normalized peak heights. The peaks lost in the presence of 3 µM KnRAV-DBD are represented by red coloring, and the peaks gained are represented by blue coloring. The horizontal axis represents the corresponding position in *proKnLBD1*. Error bars represent SD of values for three replicates.


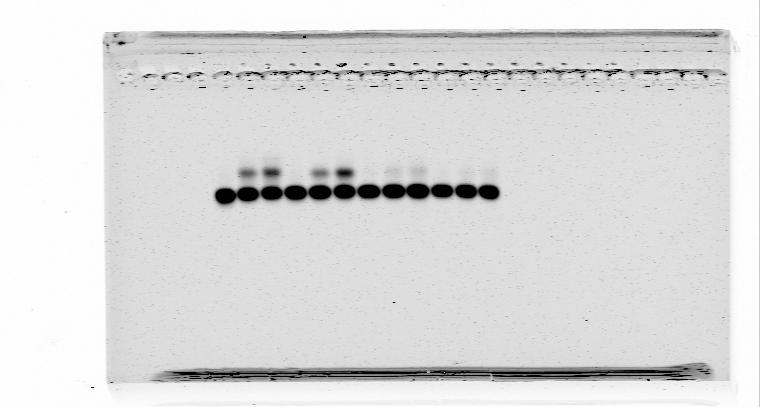
(a)


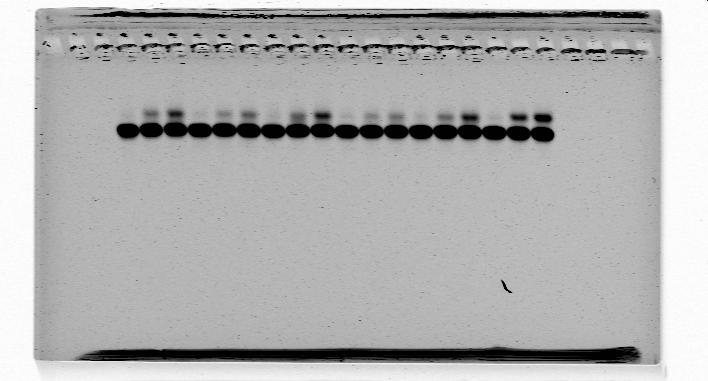
(b)

**Supplementary Figure 6. The overall image of the EMSA**.

(a) This is the full-length gel image of the EMSA shown in Figure 3c.

(b) This is the full-length gel image of the EMSA shown in Figure 3d.
